# Large Oral Lipomas: Uncommon Neoplasms in Two Case Reports

**DOI:** 10.3390/dj14040244

**Published:** 2026-04-20

**Authors:** Juraj Brozović, Bruno Vidaković, Barbara Mikulić, Matej Tomas

**Affiliations:** Faculty of Dental Medicine and Health, University of Osijek, 31000 Osijek, Croatia; bvidakovic@fdmz.hr (B.V.);

**Keywords:** lipoma, mouth neoplasms, lip, benign neoplasms

## Abstract

**Background**: Oral lipomas are uncommon benign tumors composed of mature adipocytes, accounting for roughly 1% of benign intraoral lesions. Common predilection sites are buccal mucosa, lips, and tongue, presenting as slow-growing, nodular masses, often with a yellow hue. As the size of most lesions does not exceed 10 mm, particularly larger lipomas may be misdiagnosed. We present two cases of large oral lipomas. **Case reports**: Case 1: A 58-year-old male with a painless, sessile nodular mass of approximately 2.5 cm in the left cheek, increasing in size and causing discomfort during mastication. After excision, histopathology revealed mature adipocytes with delicate fibrous septa. Case 2: A 47-year-old female with a tender yellow growth of approximately 2 cm in her lower lip, increasing in size and causing aesthetic problems with functional discomfort. After sharp dissection, the specimen presented acanthotic and parakeratotic epithelium with adipocytic tumorous tissue, permeated by collagenous cords. **Conclusions**: Oral lipomas are uncommon, mostly asymptomatic benign lesions. Mostly found in the buccal mucosa and lower lip, they can mimic more common growths. When located superficially, a conservative surgical excision leads to resolution with rare recurrences. Histopathological inspection is necessary to confirm the benign nature of the lesion.

## 1. Introduction

Adipocytic neoplasms are considered the most common soft tissue tumors, ranging from benign lipoma subtypes to locally aggressive atypical lipomatous tumors and malignant liposarcoma variants. The 2020 World Health Organization (WHO) classification subdivides benign adipocytic lesions into the following major entities: lipoma, lipomatosis, lipoblastoma, angiolipoma, myolipoma, chondroid lipoma, spindle cell lipoma, atypical spindle cell or pleomorphic lipomatous tumor and hibernoma. Lipomas (lipomata) are benign neoplasms originating from mesenchymal connective tissue and are mainly composed of mature adipocytes [1]. They can occur in all regions of the body but are most frequently found in the subcutaneous tissues of the trunk and proximal parts of the limbs. Most of these lesions are characterized as solitary, soft, well-circumscribed, painless and slow-growing, rarely exceeding 2 cm in size. Histologically, lipomas resemble their mature tissue counterparts, with an addition of a thin fibrous capsule, demarcating lobules of fat cells from the surrounding tissue [2]. The underlying pathogenesis is linked to a positive turnover balance of adipocytes, involving adipose-derived stem cells, microenvironmental factors and transcriptional differences from obesity-related fat tissue gains. Obese adipose tissue is considered hypoxic and therefore dysregulated in function, inducing upregulation of oxygen regulatory factors, metabolic adaptation, pro-inflammatory cytokines and cell necrosis, adding to the development of insulin resistance. Contrary to obese adipocytes, lipomas have a lack of their intrinsic phenomena, such as ischaemia, immune cell infiltration and pronounced glycolysis, suggesting impaired endocrine function [3].

In contrast to other locations, oral lipomas are considered uncommon neoplasms. They account for merely 0.4 to 1% of benign oral lesions [4,5,6]. The majority of cases reported involved long-lasting growths in patients above the age of 50, with females being affected slightly more. Oral lipomas have either sessile or pedunculated bases, and are well-circumscribed from the surrounding tissue, variable in color (yellow to red), rarely exceeding 10 mm in diameter. When located superficially, their distinctive yellowish color facilitates clinical diagnosis, whereas deep, infiltrating (intramuscular) specimens pose a diagnostic challenge. In deep nodular masses, salivary gland and benign mesenchymal tumors should be considered as the differential diagnoses [7,8]. When diagnostic imaging is considered, ultrasound presents very high specificity, whereas inconclusive and atypical features (i.e., deep infiltration, rapid growth, pain, size > 5 cm) indicate magnetic resonance imaging (MRI) [9,10]. The most common predilection site of an intraoral lipoma is the buccal mucosa, followed by the lips, retromolar region, tongue, vestibule, floor of the mouth and palate. Several histological subtypes were reported, predominantly conventional lipoma and fibrolipoma, differentiated by the amount and distribution of connective tissue bands. Within the capsule, lobules of mature adipocytes, varying in size and shape, are interspersed by either thin fibrous septa (classic lipoma) or broad bands of fibrocollagenous tissue (fibrolipoma). Other related entities include: myxoid lipoma, characterized by a myxoid background associated with adipose tissue, chondroid lipoma, with the addition of chondroid matrix, sialolipoma, containing atrophic salivary acini and ducts, angiolipoma, distinctive for its higher vascularity and presence of fibrin thrombi, and oral spindle cell lipoma, composed of spindle and mast cells in myxoid and collagenous tissue. Histopathological examination is essential for establishing the final diagnosis, as it is important to rule out uncommon neoplasms, such as oral liposarcoma, termed also “atypical lipomatous tumor” in its well-differentiated variant. The standard of care for all benign variants is conservative surgical excision, with similar prognosis and rare recurrences [5,11].

This paper presents two cases of oral lipoma, located in the buccal and labial mucosa, and exceeding the typical size threshold.

## 2. Case Series

### 2.1. Case Report 1—Buccal Lipoma

A 58-year-old male patient was referred for surgical management of a painless growth, located on the inner aspect of the left cheek. We noted no significant issues in the reported medical history.

**Figure 1:**
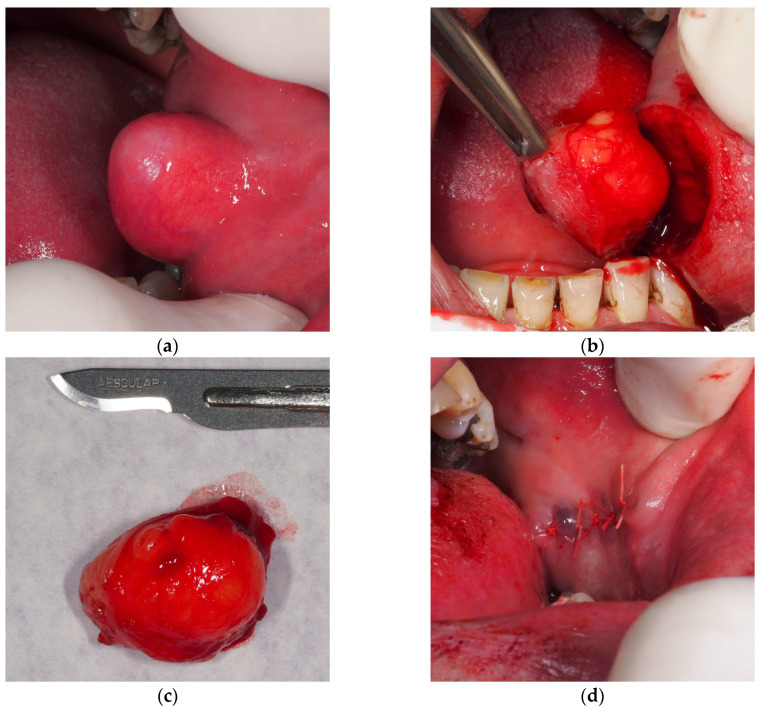
Buccal mucosa lipoma: (**a**) A sessile nodular mass in the left mid-buccal mucosa; (**b**) Excision of the lesion; (**c**) Discretely lobulated “soapy” mass; (**d**) Wound closure with PGA Resoquick fast-absorbable suture.

The patient complained of having a soft lump, which gradually increased in size over the last two years and lately caused discomfort while chewing. Extraoral clinical examination with teeth in contact revealed a discrete swelling of the left cheek. Intraorally, a sessile nodular mass of approximately 2.5 cm was found in the left mid-buccal mucosa, firm and painless to palpation, with distinctive telangiectasias in the surface lining tissue (Figure 1a).

**Figure 2:**
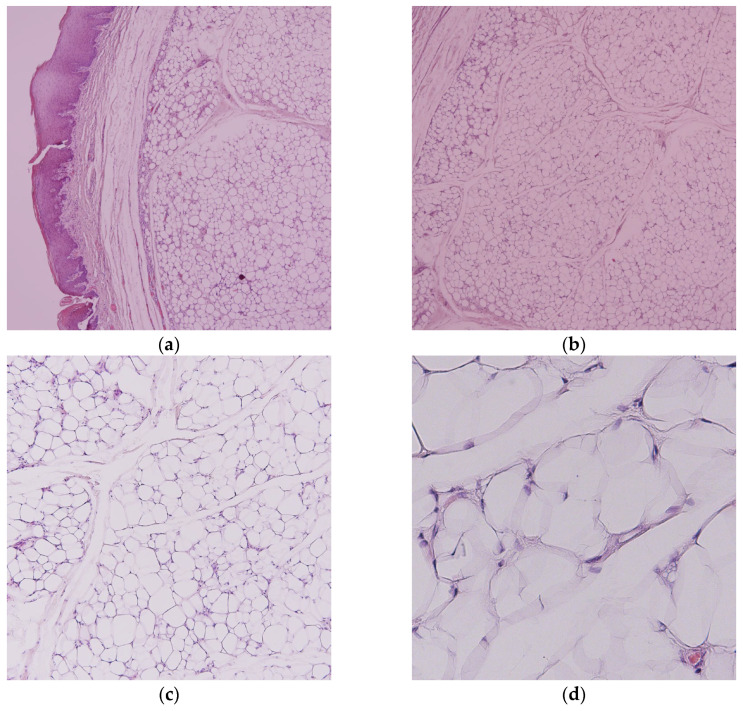
Buccal lipoma histopathology: (**a**) Incapsulated tumorous mass under stratified squamous epithelium; (**b**) Mature adipocytes permeated by delicate fibrous septa (H&E staining, 40× magnification); (**c**) 100× magnification; (**d**) 400× magnification.

We performed a complete surgical excision of the tumorous mass under local analgesia with 4% articaine hydrochloride (Ubistesin forte, 1:100,000, 3M, Neuss, Germany) by elliptical mucosal incision over the growth and blunt separation from the surrounding submucosa and muscle fibers (Figure 1b). Clinically, the round yellow mass had a “soapy” consistency and a discretely lobulated appearance (Figure 1c). The whole specimen was sent for histopathological examination. The wound closure was carried out using a fast-absorbable suture (PGA Resoquick, Resorba, Nuremberg, Germany) (Figure 1d), with uneventful healing, and no recurrence reported in four postoperative years. The pathological examination revealed a tumorous mass, composed of mature adipocytes with delicate fibrous septa, leading to the final diagnosis of oral lipoma (Figure 2a–d).

### 2.2. Case Report 2—Labial Lipoma

A 47-year-old female patient was referred for surgical treatment of lip asymmetry, due to a tender growth of approximately 2 cm in her lower lip. We noted an unremarkable medical history.

**Figure 3:**
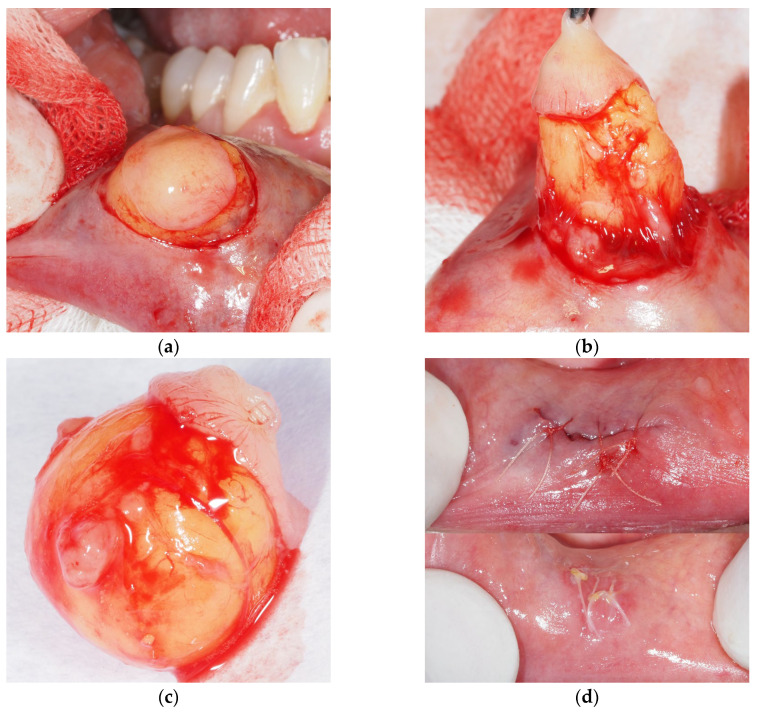
Lower lip lipoma: (**a**) A firm, broad-based, cyst-like mass with a light yellow tone; (**b**) Excision of the lesion; (**c**) A ball-shaped, cystic, “soapy” specimen, with minor salivary glands adhering to the capsule; (**d**) Wound closure with PGA Resoquick fast-absorbable suture, early healing.

The patient reported displeasing appearance and mild discomfort in the right side of her lower lip, increasing in size and intensity over the last eight months. Extraoral clinical examination confirmed swelling of the right side of the lower lip. Intraoral examination revealed a firm, broad-based, sessile mass with a light yellow tone in the overlying mucosa, with no distinction in the surface texture (Figure 3a).

**Figure 4:**
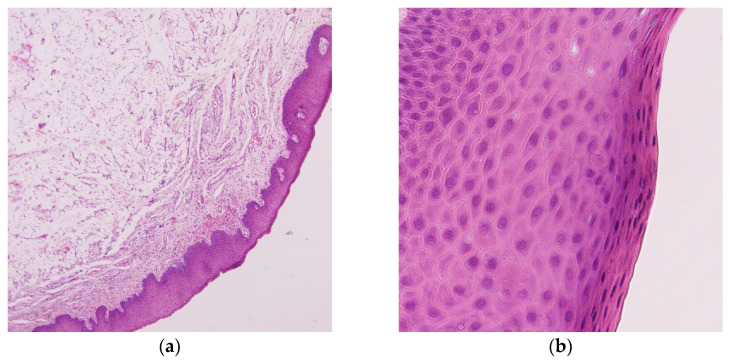

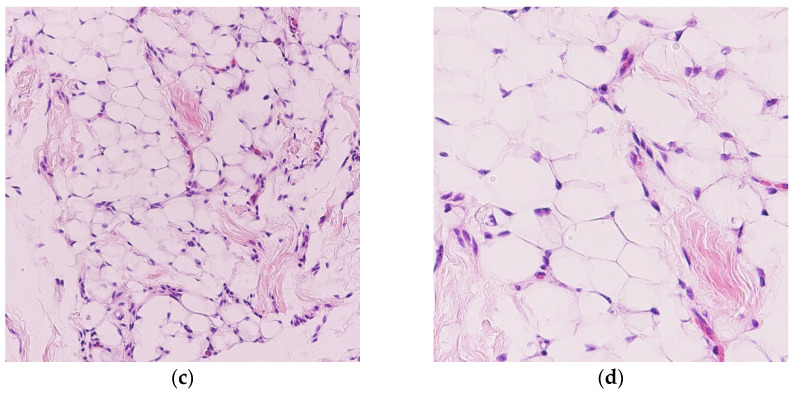
Lip lipoma histopathology: (**a**) Subepithelial tumorous tissue comprising mature adipocytes (H&E staining, 40× magnification); (**b**) Acanthotic and parakeratotic stratified squamous epithelium overlying the tumorous mass (400× magnification); (**c**) Mature adipocytes exhibiting no cellular pleomorphism, permeated by cords of collagenous tissue with scattered blood vessels (200× magnification); (**d**) 400× magnification.

After achieving local analgesia with 4% articaine hydrochloride (Ubistesin forte, 1:100,000, 3M, Neuss, Germany), we surgically excised the tumorous mass by elliptical mucosal incision over the growth, and deep sharp dissection from the neighboring tissue and muscle fibers (Figure 3b). Clinically, the growth had a ball-shaped, cystic appearance, and a firm “soapy” feel to the touch, with evident minor salivary glands adhering to the capsule (Figure 3c). The entire specimen was submitted for histopathological examination. The wound was closed with a fast-absorbable suture (PGA Resoquick, Resorba, Germany), with uneventful healing (Figure 3d), and no recurrence reported in eight postoperative months. Histopathological examination revealed acanthotic and parakeratotic stratified squamous epithelium, with tumorous tissue subepithelial, comprising mature adipocytes with no cellular pleomorphism or lipoblasts, permeated by collagenous cords of connective tissue, and a haphazard occurrence of regularly shaped blood vessels, supporting the final diagnosis of lipoma (Figure 4a–d).

## 3. Discussion

Lipomas are adipocytic neoplasms and represent the most common benign soft tissue tumors found in extremities, trunk, head and neck, retroperitoneum and mediastinum [1]. They resemble normal adipose tissue; however, they have impaired endocrine function and a different metabolic pattern. Whilst otherwise common in the subcutaneous tissues of the rest of the body, lipomas remain uncommon occurrences in the oral cavity, particularly specimens exceeding the usual dimensions. Located in the soft tissue, they grow in a circumscribed pattern at a slow pace, and are often unnoticed until aesthetics, mastication or speech become impaired, as was the case with the patients described in this report. The literature reviews found that most intraoral lipomas were less than 10 mm in diameter [8,12], with average sizes reported as approximately 1.3 cm [4], 1.6 cm [5], and 2.1 cm [6]. Above the 1.8–2 cm threshold, lesions in case reports were deemed as “unusual” and “large” [13,14,15,16]. Hence, the lipoma specimens presented in this paper fall within this category, exceeding 2 cm in size, in particular the buccal variant, presenting similar to that reported by De Sanctis et al. [14]. Regarding location, lipomas of the buccal mucosa are the most commonly reported, making up roughly 1/3 of the cases, followed by tongue lipomas (1/4 of the cases), and lip lipomas (1/10 of the cases) [8]. Some studies reported that the lower lip is affected more frequently than the tongue, regarding it as the second most frequent site [11,16,17]. Therefore, apart from the size, the two cases presented here could be considered representative for the oral region. In the lower lip case, the distinctive preoperative feature was the light yellow hue in the overlying mucosa, making it distinguishable from more common lip lesions. A retrospective study by Barros et al. [18] reported that mucoceles and vascular lesions were the most common masses found in this area, whereas less frequent resembling conditions like oral angioedema and lingual lymphangioma have also been described [19,20]. Superficially located variants must also be differentiated from oral dermoid, epidermoid and lymphoepithelial cysts [16]. In the buccal lipoma case, there was no such distinctive hue in the mucosa; however, during the excision, the underlying mass showed a characteristic yellow color. This mucosal area is otherwise frequently exposed to local irritation, and knob-like nodular growths in the buccal mucosa are usually diagnosed as fibro-epithelial polyps, the most common benign soft tissue growth in the mouth [21]. The differential diagnosis often includes mucocele, giant cell lesions, papillomas, neoplasms, and, as in our case, lipomas [22]. Several theories have been proposed to explain the occurrence of buccal lipomas: continuous trauma, hypertrophy, and connective tissue metaplasia [15]. In addition to these pathogenetic theories, it is also hypothesized that these masses might occur by inward hernation of the buccal fat pad [23,24]. Our case revealed no such obvious prolapse, through the buccinator muscle, favoring the first two theses.

Histopathological examination is considered the gold standard for establishing the final diagnosis, particularly in ruling out possible malignancies. Histologically, oral lipoma variants are usually classified as conventional lipoma, followed by fibrolipoma, less common spindle cell lipoma, sialolipoma, chondrolipoma, and rarely reported, angiofibrolipoma [23]. The ratio of lipomas to fibrolipomas in the literature is very variable, ranging from 1:1 to less than 1:10 [6,25,26,27]. The histology in both of our specimens did not show abundant fibrous stroma; rather, it demonstrated delicate fibrous septa between adipocytes, identifying them as lipomas. Regardless of the subtype, conservative local excision is the standard of care. Recurrence is rare and is more associated with an incomplete surgical excision of intramuscular lipomas, where a wider surgical excision might be preferred [6,28,29]. There were no muscle fibers detected in either of our specimens, which is consistent with the superficial submucosal subtype. Apart from incomplete surgical excision, De Sanctis et al. [14] indicated that an aggressive excision might lead to a perforation in its capsule, causing spillage of cells into the surrounding tissue, thus increasing the risk of recurrence. In both of our cases, we performed a conservative local excision, with no sign of recurrence after four years (buccal lipoma) and after ten months (labial lipoma).

## 4. Conclusions

Oral lipomas are uncommon benign neoplasms, often mimicking the appearance of more common soft tissue growths, particularly in the buccal and labial sites. The history of slow growth, low-level discomfort and the clinical finding of a sessile nodular mass with a distinctive yellow hue in the overlying mucosa are suggestive of lipoma. When located superficially, a conservative surgical excision leads to resolution with rare recurrences. Deeper, infiltrating lesions often require wider excision margins. Despite the benign nature of lipomas and similar growths in buccal and labial sites, histopathological examination is essential for confirming the benign nature of the lesion.

## Data Availability

The original contributions presented in this study are included in the article. Further inquiries can be directed to the corresponding authors.

## References

[1] Bansal A., Goyal S., Goyal A., Jana M. (2021). WHO classification of soft tissue tumours 2020: An update and simplified approach for radiologists. Eur. J. Radiol..

[2] Dalal K.M., Antonescu C.R., Singer S. (2008). Diagnosis and management of lipomatous tumors. J. Surg. Oncol..

[3] Suga H., Eto H., Inoue K., Aoi N., Kato H., Araki J., Higashino T., Yoshimura K. (2009). Cellular and molecular features of lipoma tissue: Comparison with normal adipose tissue. Br. J. Dermatol..

[4] Perez-Sayáns M., Blanco-Carrión A., Oliveira-Alves M.G., Almeida J.D., Anbinder A.L., Lafuente-Ibáñez De Mendoza I., Aguirre-Urízar J.M. (2019). Multicentre retrospective study of 97 cases of intraoral lipoma. J. Oral Pathol. Med..

[5] Pires F.R., Souza L., Arruda R., Cantisano M.H., Picciani B.L., Dos Santos T.C. (2021). Intraoral soft tissue lipomas: Clinicopathological features from 91 cases diagnosed in a single Oral Pathology service. Med. Oral.

[6] Juliasse L.E.R., Nonaka C.F.W., Pinto L.P., Freitas R.D.A., Miguel M.C.D.C. (2010). Lipomas of the oral cavity: Clinical and histopathologic study of 41 cases in a Brazilian population. Eur. Arch. Otorhinolaryngol..

[7] Matiakis A., Karakostas P., Mylonas A.I., Anagnostou E., Poulopoulos A. (2020). Oral lipoma: A clinicopathological study of 37 cases and a brief review of the literature. Hell. Arch. Oral Maxillofac. Surg..

[8] Egido-Moreno S., Lozano-Porras A., Mishra S., Allegue-Allegue M., Mari-Roig A., Lopez-Lopez J. (2016). Intraoral lipomas: Review of literature and report of two clinical cases. J. Clin. Exp. Dent..

[9] Johnson C.N., Ha A.S., Chen E., Davidson D. (2018). Lipomatous Soft-tissue Tumors. J. Am. Acad. Orthop. Surg..

[10] Rahmani G., McCarthy P., Bergin D. (2017). The diagnostic accuracy of ultrasonography for soft tissue lipomas: A systematic review. Acta Radiol. Open.

[11] Osterne R.L.V., Lima-Verde R.M.B., Turatti E., Nonaka C.F.W., Cavalcante R.B. (2019). Oral cavity lipoma: A study of 101 cases in a Brazilian population. J. Bras. Patol. Med. Lab..

[12] Kumaraswamy S., Madan N., Keerthi R., Shakti S. (2009). Lipomas of oral cavity: Case reports with review of literature. J. Maxillofac. Oral Surg..

[13] Dhinoja K.N. (2024). Intraoral Lipoma. Adv. Hum. Biol..

[14] De Sanctis C.M., Zara F., Sfasciotti G.L. (2020). An Unusual Intraoral Lipoma: A Case Report and Literature Review. Am. J. Case Rep..

[15] Bonavolontà P., Togo G., Tarallo G., Abbate V., Maffia F., Sarcinella M., Spinelli R., Orabona G.D., Califano L. (2023). Large intraoral Lipoma: A case report of rare neoformation of the lower lip. Oral Maxillofac. Surg. Cases.

[16] Mehendirratta M., Jain K., Kumra M., Manjunatha B.S. (2016). Lipoma of mandibular buccal vestibule: A case with histopathological literature review. BMJ Case Rep..

[17] Karakostas P., Matiakis A., Anagnostou E., Kolokotronis A. (2018). Oral lipoma located at the left lower vestibule-report of a case and a brief review of the literature. Balk. J. Dent. Med..

[18] Barros C.C., Medeiros C.K., Rolim L.S., Cavalcante I.L., Santos P.P., Silveira É.J., Oliveira P.T. (2020). A retrospective 11-year study on lip lesions attended at an oral diagnostic service. Med. Oral.

[19] De Falco D., Misceo D., Carretta G., Gioco G., Lajolo C., Petruzzi M. (2025). Oro-Facial Angioedema: An Overview. Immuno.

[20] De Falco D., Ingravallo G., Petruzzi M. (2025). Frog Egg Tongue. AIM Clin. Cases.

[21] Mishra A., Pandey R.K. (2016). Fibro-epithelial polyps in children: A report of two cases with a literature review. Intractable Rare Dis. Res..

[22] Bhuvaneswari B., Catherine J., Vikram V., Jagannathan R., Balaji T.M. (2020). A bizarre presentation of fibroepithelial polyp—A case report. J. Oral Dis. Marker.

[23] Agha-Hosseini F., Moslemi E. (2014). Angiofibrolipoma of the retromolar pad region: Case report. New York State Dent. J..

[24] Venkateswarlu M., Geetha P., Srikanth M. (2011). A rare case of intraoral lipoma in a six year-old child: A case report. Int. J. Oral Sci..

[25] Trandafir D., Gogălniceanu D., Trandafir V., Căruntu I.D. (2007). Lipomas of the oral cavity—A retrospective study. Rev. Med. Chir. Soc. Med. Nat. Iasi.

[26] Furlong M.A., Fanburg-Smith J.C., Childers E.L.B. (2004). Lipoma of the oral and maxillofacial region: Site and subclassification of 125 cases. Oral Surg. Oral Med. Oral Pathol. Oral Radiol. Endod..

[27] Fregnani E.R., Pires F.R., Falzoni R., Lopes M.A., Vargas P.A. (2003). Lipomas of the oral cavity: Clinical findings, histological classification and proliferative activity of 46 cases. Int. J. Oral Maxillofac. Surg..

[28] Chidzonga M.M., Mahomva L., Marimo C. (2006). Gigantic tongue lipoma: A case report. Med. Oral Patol. Oral Cir. Bucal.

[29] Piattelli A., Fioroni M., Rubini C. (2000). Intramuscular lipoma of the cheek: A case report. J. Oral Maxillofac. Surg..

